# A robust multiple-objective decision-making paradigm based on the water–energy–food security nexus under changing climate uncertainties

**DOI:** 10.1038/s41598-021-99637-7

**Published:** 2021-10-22

**Authors:** Maedeh Enayati, Omid Bozorg-Haddad, Elahe Fallah-Mehdipour, Babak Zolghadr-Asli, Xuefeng Chu

**Affiliations:** 1 Department of Irrigation and Reclamation Engineering, Faculty of Agricultural Engineering and Technology, College of Agriculture and Natural Resources, University of Tehran, 3158777871 Karaj, Iran; 2 Department of Water and Energy, Moshanir Consultant Co., Tehran, Iran; 3 Department of Civil, Construction and Environmental Engineering, North Dakota State University, Dept 2470, Fargo, ND 58108-6050 USA

**Keywords:** Ecology, Climate sciences, Ecology, Environmental sciences, Environmental social sciences, Hydrology, Natural hazards, Energy science and technology, Engineering, Mathematics and computing

## Abstract

From the perspective of the water–energy–food (WEF) security nexus, sustainable water-related infrastructure may hinge on multi-dimensional decision-making, which is subject to some level of uncertainties imposed by internal or external sources such as climate change. It is important to note that the impact of this phenomenon is not solely limited to the changing behavior patterns of hydro-climatic variables since it can also affect the other pillars of the WEF nexus both directly and indirectly. Failing to address these issues can be costly, especially for those projects with long-lasting economic lifetimes such as hydropower systems. Ideally, a robust plan can tolerate these projected changes in climatic behavior and their associated impacts on other sectors, while maintaining an acceptable performance concerning environmental, socio-economic, and technical factors. This study, thus, aims to develop a robust multiple-objective decision-support framework to address these concerns. In principle, while this framework is sensitive to the uncertainties associated with the climate change projections, it can account for the intricacies that are commonly associated with the WEF security network. To demonstrate the applicability of this new framework, the Karkheh River basin in Iran was selected as a case study due to its critical role in ensuring water, energy, and food security of the region. In addition to the *status quo*, a series of climate change projections (i.e., RCP 2.6, RCP 4.5, and RCP 8.5) were integrated into the proposed decision support framework as well. Resultantly, the mega decision matrix for this problem was composed of 56 evaluation criteria and 27 feasible alternatives. A TOPSIS/Entropy method was used to select the most robust renovation plan for a hydropower system in the basin by creating a robust and objective weighting mechanism to quantify the role of each sector in the decision-making process. Accordingly, in this case, the energy, food, and environment sectors are *objectively* more involved in the decision-making process. The results revealed that the role of the social aspect is practically negligible. The results also unveiled that while increasing the power plant capacity or the plant factor would be, seemingly, in favor of the energy sector, if all relevant factors are to be considered, the overall performance of the system might resultantly become sub-optimal, jeopardizing the security of other aspects of the water–energy–food nexus.

## Introduction

Principally, the sustainable management of water resources can be considered as one of the key features for agricultural practices. Thus, it would be reasonable to assume that achieving *food security* for a community, in practice, is tied to the security of water resources. It is also apparent that survival and, in turn, the prosperity of a society depend heavily on food security. Nonetheless, water resources management plans laid solely with respect to the agriculture/food sector or without consideration of the intricately intertwined connection between the water–energy–food (WEF) sectors actively dismiss the multi-dimensional nature of water and, in turn, neglect the rest of stakeholders such as the environmental sector. In fact, ever since the 1960s, environmental concerns have gained momentum in the pop culture, up to the point that preserving natural ecosystems, maintaining biodiversity, and sustaining the environment have been profusely perused by these environmentally aware communities^1^. As a result, environment-oriented objectives have become integral aspects of modern water resources management frameworks.

On the other hand, water also plays a crucial role in achieving *energy security*. Hydropower is considered as a clean, renewable energy resource that, under the right circumstances, can maintain a certain level of economic growth while minimizing the environmental degradation by controlling the communities’ dependency on fossil fuels^2^. The main challenge here is that these hydropower systems, especially as they are scaled up, are exposed to some degree of risk imposed by fluctuations in hydro-climatic variables. One of the most notable and controversial sources for these projected fluctuations is the *climate change* phenomenon. Gaudard and Romerio^3^, for instance, projected the impact of climate change on the Europe’s hydropower industry. They concluded that while the hydropower industry has a promising future indeed, such a risk should not be in any way underestimated.

From a regulatory perspective, in most countries, the first priority in water resources management is to meet the *drinking* water *needs* of any given community. Although this approach toward water resources planning and management may make sense when facing water shortages, resorting to such a viewpoint would potentially undermine the whole notion of the WEF security nexus, as it essentially creates a hierarchy for water consumption sectors. Undoubtedly, a wholesome plan should not separate the three pillars of WEF and, in turn, address their intertwined connection to ensure a sustainable solution for all WEF sectors. Resultantly, the complex interactions and interdependency of the water, energy, and food sectors introduced the concept of *WEF security nexus*^4,5^. This security nexus intertwines socio-economic, environmental, and technical factors, and thus, addressing this matter in terms of water resources planning and management requires a multi-criteria decision-making framework.

The core element in the above-mentioned nexus (i.e., water) is heavily dependent on the climate conditions, and any projected changes in the climate’s *status quo* can potentially influence the dynamic relationship of the elements of this security nexus. In other words, from a water resources management standpoint, any sustainable water plans must address the complex intertwined relationship of the WEF security nexus and environmental agendas. Such plans should also have the capacity of handling the uncertainties associated with the climate change phenomenon. Emphasizing solely on water demands in a conventional prioritized hierarchy structure and/or neglecting the potential impacts of climate change could result in long-term adverse impacts not only in the water sector but also in all industries that are related to any of the three pillars of the WEF nexus. Needless to say, these projections for climatic behavior patterns have the potential to affect all the pillars of the WEF security nexus either directly or in an indirect manner. Hence, it is important to note that such projections are by nature bound to some degree of uncertainty; thus, assuming a stationary governing condition while assessing this security nexus can often be far-fetched from reality^6^. Resultantly, when it comes to water resources planning and management, a *sustainable* future may hinge on a presumable decision-making process that not only addresses the multi-dimensionality of WEF nexus but also promotes *robust* solutions that can more or less tolerate these projected changes. Failing to address these matters, on the other hand, can often inadvertently create sub-optimal signals to economy, national security, and/or environment^7^. Over the years, some alternative robust decision-making frameworks have been proposed for water resources planning and management [e.g.,^6,8,9^]. The multi-attribute decision-making (MADM) framework, meanwhile, has time and again proven to be a reliable option to find a robust solution in a multi-dimensional decision space, even when some elements of the problem are exposed to some degree of uncertainty^10^.

Electricity production, one of the critical features in providing energy security, has experienced a whopping 72% increase from 1993 to 2010; according to the projections, an additional 56% raise for electricity production can still be expected by 2040^11^. Based on the *status quo*, these projections can be considered as an alarming signal for the global greenhouse gas emissions^12^. Thus, a shift toward clean and renewable energy resources (e.g., hydropower systems) and promoting such technologies on a global scale have a high priority^11^. In the meantime, when it comes to improving the state of hydropower systems, the most budget-friendly approach to address this matter is often associated with adding capacity at existing hydropower schemes or capturing energy from existing dams that have not been equipped with any hydropower facilities^13^. It should be noted that such water infrastructure could have a very long lifetime, which can even be 100 years. As such, basing these system upgrades solely on the baseline conditions and in turn ignoring the projected changes in the climate conditions can be costly in the long run^2,13^. In other words, opting for a robust solution for upgrading these systems would be crucial. While making these decisions, one must also take into consideration the WEF security nexus, which in turn converts the situation into a multi-dimensional decision-making problem. This study, thus, aims to develop an improved MADM-based framework to address these concerns. In order to demonstrate the applicability of this new framework, the Karkheh River basin in Iran is selected as a case study due to its permanent role in ensuring water, energy, and food security of the region.

## Material and methods

**Figure 1:**
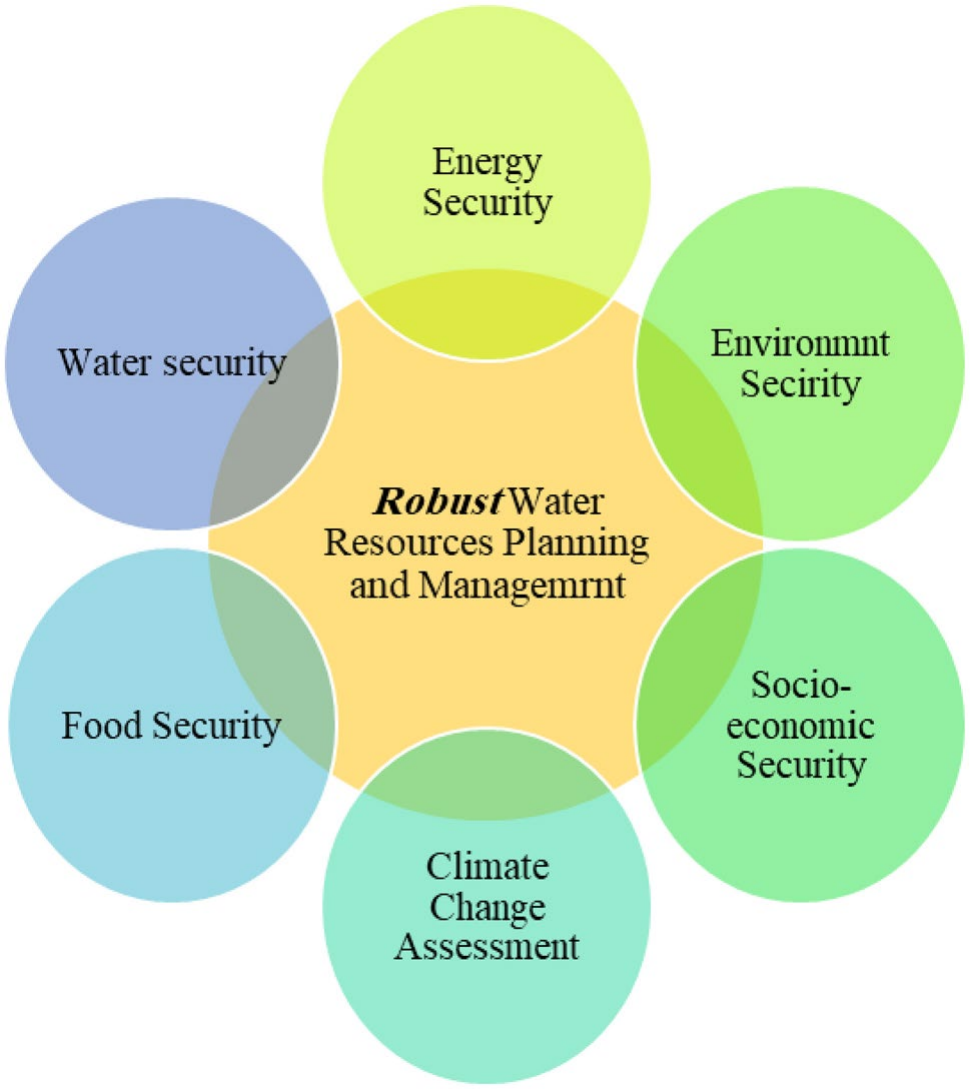
Basic components of the robust decision-making paradigm for water resources planning and management.

As stated, the primary goal of this study is to promote an objective decision support framework for water resource planning and management purposes within the context of the WEF security nexus, which takes into account the uncertainties imposed by the climate change phenomenon. Such a framework is “robust” since it takes the multi-dimensionality of water-related problems into account while addressing the uncertainties imposed by climate change projections. The basic components of this decision-making paradigm are depicted in Fig. 1. In principle, while this framework is sensitive to the uncertainties associated with the climate change projections, it can provide a dynamic water resources planning and management scheme promoted within the WEF security network. Thus, in addition to the *status quo*, a series of climate change projections (i.e., RCP 2.6, RCP 4.5, and RCP 8.5) are also integrated into the proposed decision support framework. In essence, the main components of the proposed framework are simulation and operation of the water resources system based on the standard operation policy (SOP), evaluating the system’s efficiency through a series of quantitative performance criteria, and finally, applying the MADM-based framework to opt for a robust system renovation setting.

### Simulating the water resources system

SOP is a primitive, and perhaps the most-well-known real-time operation policy in water resources planning and management^14^. The core principle here is to minimize the prioritized water shortage at the current time step with no conservation policy (e.g., hedging rules) in place. SOP, as a standard rule curve (RC), determines how the operator should behave at any given state of a reservoir^15,16^. This rule curve is established as an attempt to balance various water demands including but not limited to flood control, hydropower, water supply, and recreation^17^. A SOP operating system attempts to release water to meet a water demand at the current time, with no regard to the future.

In general, SOP can be mathematically expressed as^18^:

1a $$R_{t} = \left\{ {\begin{array}{*{20}c} {D_{t} } \\ {AW_{t} } \\ 0 \\ \end{array} - S_{\min } } \right.\begin{array}{*{20}c} {} & {if} & {AW_{t} > S_{\min } } \\ {} & {if} & {AW_{t} > S_{\min } } \\ {} & {if} & {AW_{t} \le S_{\min } } \\ \end{array} \begin{array}{*{20}c} {} & {and} & {AW_{t} - S_{\min } \ge D_{t} } \\ {} & {and} & {AW_{t} - S_{\min } < D_{t} } \\ {} & {} & {} \\ \end{array} \quad t = { 1},{ 2},{ 3}, \, ... \, ,T$$

where

1b $$AW_{t} = S_{t} + Q_{t} - Loss_{t}$$

in which *R*_*t*_ = amount of water supplied during the *t*th time step; *D*_*t*_ = consumers’ water demand during the *t*th time step; *AW*_*t*_ = amount of available water during the *t*th time step; *S*_*t*_ = amount of stored water during the *t*th time step; *S*_min_ = dead storage of the reservoir; *Q*_*t*_ = inflow during the *t*th time step; *Loss*_*t*_ = net water loss (i.e., precipitation minus evaporation) of the reservoir during the *t*th time step; and *T* = total number of time steps in the operational horizon.

In practice, however, a different type of water demand leads to a different interpretation of water shortage. There are cases in which the stakeholders’ needs are represented by a set of volumetric *demand targets*, and the decision-makers’ objective would be to minimize the water deficit based on a set of priorities for these demands. This is a typical case for agricultural, domestic, industrial, and environmental demands. For hydropower generation, however, a conventional interpretation of SOP would be to generate maximum electricity permitted by the power plant capacity (PPC) at each given time step^19^. For a hydropower system, the amount of water needed to reach a power plant capacity is given by^19^:

2 $$R_{t} = \frac{{86400 \times PF \times Countday_{t} \times PPC}}{{\gamma_{w} \times g \times \eta \times \Delta H_{t} }}$$

in which, *γ*_*w*_ = water specific weight; *g* = gravitational acceleration; *η* = efficiency of the hydropower system; Δ*H*_*t*_ = height difference between the reservoir water level and the tailwater level at time step *t*; *Countday*_*t*_ = number of days within time step *t*; and *PF* = plant factor of the hydropower system.

As stated earlier, applying an SOP-based plan requires a set of pre-defined priorities to advise decision-makers concerning the order, in which each of these demands is to be met. The major water demands include drinking, industry, environment, agriculture, and hydropower. Thus, according to the SOP’s principle, the decision-makers, first, allocate the available water to meet the demand of the stakeholder with the highest priority (i.e., the domestic and industrial demand). After this first water demand is fully satisfied, the available water can be used for the next demand. Such an allocation process continues until no water is available. It should be noted, however, that if the released water in each stage passes through the penstock equipped with the turbines, electricity can be generated. The amount of energy generated in previous stages must be accounted for before computing the amount of water released for hydropower purposes.

### Performance criteria

Performance criteria are, in essence, quantitative measures that can provide a practical insight for the decision-makers regarding the status of a system. This definition covers a broad spectrum of mathematical representations, which can range from simple mathematical formulas such as the average of a specific output to more complex and probability-based entities^20,21^. The most fundamental and universal probability-based performance criteria are *reliability*, *resiliency*, and *vulnerability*^22–24^. In essence, reliability is the probability of successful function of a system; resiliency measures the probability of successful functioning following a system failure; and vulnerability quantifies the severity of failure during an operation horizon^25^. It should be noted that these three criteria assess different aspects of a water resources system, and as such, they complement one another^26^. For more information regarding these probabilistic performance criteria, the readers can refer to Sandoval-Solis et al.^27^ and Zolghadr-Asli et al.^20^.

In this study, the concept of levelized cost of energy (LCOE) is utilized for economic evaluation. The LCOE of a given hydropower system is the ratio of lifetime costs to lifetime electricity generation, both of which are discounted back to a common year using a discount rate that reflects the average cost of capital^28^. The LCOE of renewable energy systems depends on the technology, geographic criteria, capital and operating costs, and the efficiency of the system. The LCOE can be mathematically expressed as follows^29^:

3 $$LCOE = \frac{{\sum\nolimits_{t = 1}^{n} {\frac{{I_{t} + M_{t} + F_{t} }}{{\left( {1 + r} \right)^{t} }}} }}{{\sum\nolimits_{t = 1}^{n} {\frac{{E_{t} }}{{\left( {1 + r} \right)^{t} }}} }}$$

in which *I*_*t*_ = investment expenditures in year *t*; *M*_*t*_ = operation and maintenance expenditures in year *t*; *F*_*t*_ = fuel expenditures in year *t*; *E*_*t*_ = electricity generation in year *t*; *r* = discount rate; and *n* = economic life expectancy of the system.

### MADM

MADM is an umbrella term to describe a series of frameworks, which aim to help individuals or a group of individuals to prioritize a series of discretely defined alternatives with regard to a set of evaluation attributes^30,31^. MADM can provide the necessary means to conduct planning and management under changing circumstances such as those under climate change conditions^10,32^. According to one of the basic principles of MADM, the decision-maker can use the similarity of the feasible alternatives and the preferential result and/or incongruity of the undesirable alternatives. The notion mentioned above is, chiefly, the core principle of the reference-dependent theory^33^. Accordingly, the reference-based branch of the MADM methods can, itself, be classified into two major groups: *screening methods* and *ranking methods*. Screening methods eliminate alternatives that cannot satisfy the pre-determined conditions for the desirable solution, while ranking methods order all the alternatives from the best to the worst^34^.

Pioneered by Hwang and Yoon^35^, the technique for order references by similarity to an ideal solution (TOPSIS) is a *compensatory*, *objective* MADM solving method rooted from the basic principles of the reference-dependent theory. The core idea is that the chosen alternative should have the shortest distance from the *ideal solution* and the farthest distance from the *negative-ideal solution*^36^. The basic computation algorithm of TOPSIS can be summarized as follows^37,38^:

Step I: Construct the original decision matrix (*X*), where *m* feasible alternatives are to be evaluated based on *n* evaluation criteria:

4 $$X = \left[ {\begin{array}{*{20}c} {x_{11} } & {x_{12} } & \cdots & {x_{1n} } \\ {x_{21} } & {x_{22} } & \cdots & {x_{2n} } \\ \vdots & \vdots & \ddots & \vdots \\ {x_{m1} } & {x_{m2} } & \cdots & {x_{mn} } \\ \end{array} } \right]$$

in which *x*_*ij*_ = the element of the *i*th alternative concerning the *j*th criterion.

Step II: Defining the reference alternatives [i.e., the ideal solution (*s*^+^) and the negative-ideal solution (*s*^−^)]. To do so, first, the elements of the decision matrix that are associated with *negative criteria* must be redefined by using the following equation:

5a $$x_{ij}^{ * } = \frac{1}{{x_{ij} }}$$

The elements of the decision matrix that are associated with *positive criteria* would remain the same:

5b $$x_{ij}^{ * } = x_{ij}$$

The ideal alternative is an arbitrarily defined vector, which describes the aspired solution to the given problem, while the inferior alternative is an arbitrarily defined solution that represents the most undesirable option for the given MADM problem. Here, the ideal and negative-ideal solutions would be represented with two separate vectors where each pair of the corresponding elements in these vectors is, respectively, the *maximum* and *minimum* values of $x_{ij}^{ * }$ with regard to each of the evaluation criteria.

Step III: Each element of the decision matrix should be *normalized* by using the following equation:

6 $$p_{ij} = \frac{{x_{ij}^{ * } }}{{\sqrt {\sum\nolimits_{i = 1}^{m} {x_{ij}^{ * 2} } } }}$$

in which *p*_*ij*_ = the normalized performance value for the *i*th alternative with respect to the *j*th criterion.

Step IV: The weighted normalized preference value (*z*_*ij*_) can be computed as follows:

7 $$z_{ij} = p_{ij} \times w_{j} \quad \forall i,j$$

in which *w*_*j*_ = the weight (i.e., the importance value) of the *j*th criterion. The weights assigned to the evolution criteria reflect their relative importance to the decision-makers. The higher the weights are, the more crucial their roles would be in the selection process. Chiefly, these weighting mechanisms are either *subjective* in nature or follow an *objective* procedure. In the subjective approaches, the weights of the attributes are assigned based on the performance information given by the decision-maker, whereas in the objective approaches, the weights of the evaluation attributes would be obtained by using the objective information extracted from the decision matrix^39^. Shannon’s Entropy method, used in this study as the weight assignment mechanism, is a well-known objective weighting technique^40^. This method tends to assign the highest weight to an evaluation attribute with the highest dispersity in its values. For more information on the computational framework of this method, the readers can refer to Lotfi and Fallahnejad^41^.

Step V: In this step, every given alternative is compared to the reference points, namely, the ideal and inferior alternatives. The described procedure, which is known as the separation measurement in TOPSIS, can be mathematically expressed as follows^35^:

8 $$D_{i}^{ + } = \sqrt {\sum\limits_{j = 1}^{n} {\left( {z_{ij} - z_{j}^{ + } } \right)^{2} } }$$

And

9 $$D_{i}^{ - } = \sqrt {\sum\limits_{j = 1}^{n} {\left( {z_{ij} - z_{j}^{ - } } \right)^{2} } }$$

in which $D_{j}^{ + }$ and $D_{j}^{ - }$ = separation measurements of the *j*th criterion with respect to the ideal and inferior alternatives, respectively.

Step VI: The relative closeness to the ideal solution (*χ*_*i*_), which can be used to rank the desirability of the feasible alternative, can be computed as follows^35^:

10 $$\chi_{i} = \frac{{D_{i}^{ - } }}{{D_{i}^{ + } + D_{i}^{ - } }}\quad \forall i$$

The further this distance (i.e., larger values of *χ*_*i*_), the more desirable the alternative would be.

### Robust multi-attribute framework

As stated, each climate change scenario depicts a unique future with regard to the changing climate, which in turn introduces an element of uncertainty to the projected performance of water resources systems during their operation horizon. Furthermore, downscaling methods, which link these projected changes in the global climatic pattern to a local or regional scale, can be another source of uncertainty. Naturally, for long-lasting water infrastructure such as a hydropower system, addressing these uncertainties in a proper and timely manner can be one of the key components of a robust project. Thus, this study aims to not only evaluate the system’s performance under the *status quo* but also assess the credibility of the system under the projected *climate change* conditions.

**Figure 2:**
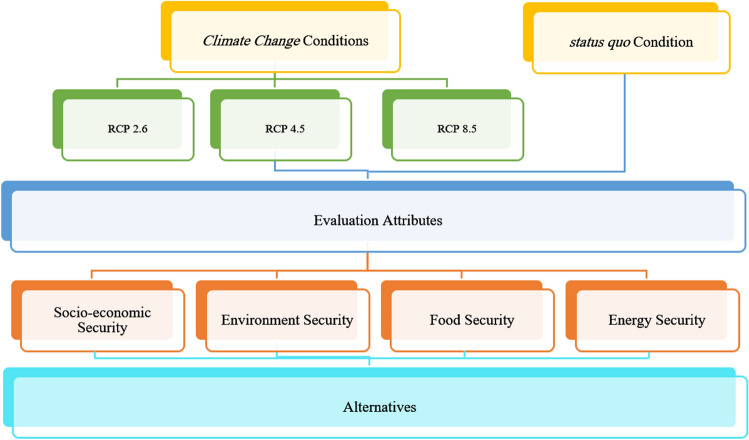
Schematic diagram of the MADM problem.

**Figure 3:**
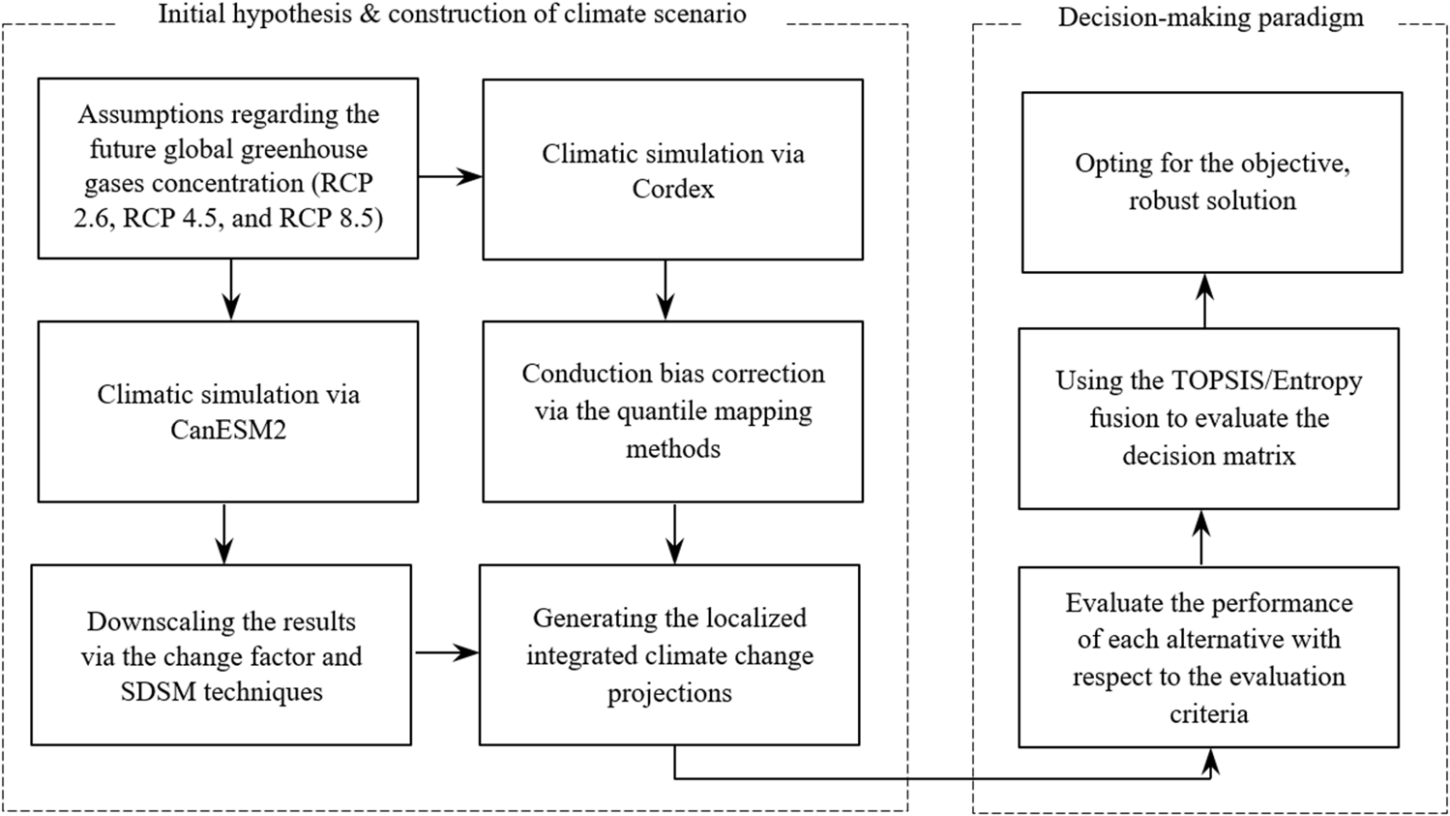
Flowchart of the proposed framework.

The other characteristic one might expect from a robust project is its ability to take into account the multi-dimensionality nature of water-related infrastructure. Most notably, addressing the WEF security nexus must be a priority in water resources planning and management. Resultantly, any robust decision-making paradigm for water resources planning and management purposes should also account for the other pillars of the WEF nexus (i.e., energy and food sectors), as they would be consequentially affected by such decisions. It is also important to note that these sectors could be affected by the climate change phenomenon. The other crucial feature of a robust decision-making paradigm is that it should be able to account for the socio-economic, environmental, and technical factors that determine the overall quality of the project. Such a decision-making paradigm is depicted in Fig. 2. This notion in practice, however, can typically lead to a mega decision matrix composed of numerous criteria and alternatives that can be overwhelming if the subjective MADM methods are to be employed. This study, thus, employs an objective MADM framework (i.e., TOPSIS/Entropy) to help overcome the above-described problem. The basic idea is to promote a universal and practical decision support framework that enables the water resources planners and managers to account for the intricacies of the WEF security nexus while simultaneously taking the uncertainties of climate change projections into account. Figure 3 illustrates the flowchart of the proposed decision support framework.

## Case study

The Karkheh River basin, with an area of 51,000 km^2^, is located in the southwest of Iran. The basin’s strategic role in water and food security in the country has earned it the nickname: Iran’s *food basket*. Furthermore, the basin’s immense potential for hydropower has encouraged the decision-maker to actively pursue and promote new projects to harness these renewable energy resources. Thus, any practical long-term plans for the water resources of the Karkheh River basin must, in a way, address the WEF security nexus.

Typically, the Karkheh River basin experiences high spatiotemporal variations in precipitation and air temperature^42^. Accordingly, the southern part of the basin receives an average annual precipitation of approximately 150 mm, while the average annual precipitation in the northern and northeastern parts is reported to be as high as 1000 mm. The air temperature in the basin ranges from − 25 to 50 °C. Overall, the southern area of the catchment can be classified as semi-arid with mild winters and long hot summers, while the northern part and the alpine regions have cold winters and mild summers. As a result, both rain-fed and irrigated agriculture are practiced in the northern part of the basin, while irrigated agriculture is solely practiced in the southern part of the basin due to its arid climate^42,43^.

The Karkheh River, which is originated from the Zagros Mountains in Western Iran, is approximately 900 km long and discharges into the Hoor-Al-Azim swamp at the Iran-Iraq border. Based on the available datasets, the average annual discharge of the Karkheh River under the baseline condition is about 5916 million m^3^ (MCM). In addition to its permanent role in the energy and agriculture sectors, the Karkheh River also plays a crucial part in establishing the downstream ecosystem and supplying water for the domestic needs of the nearby communities^43^.

**Figure 4:**
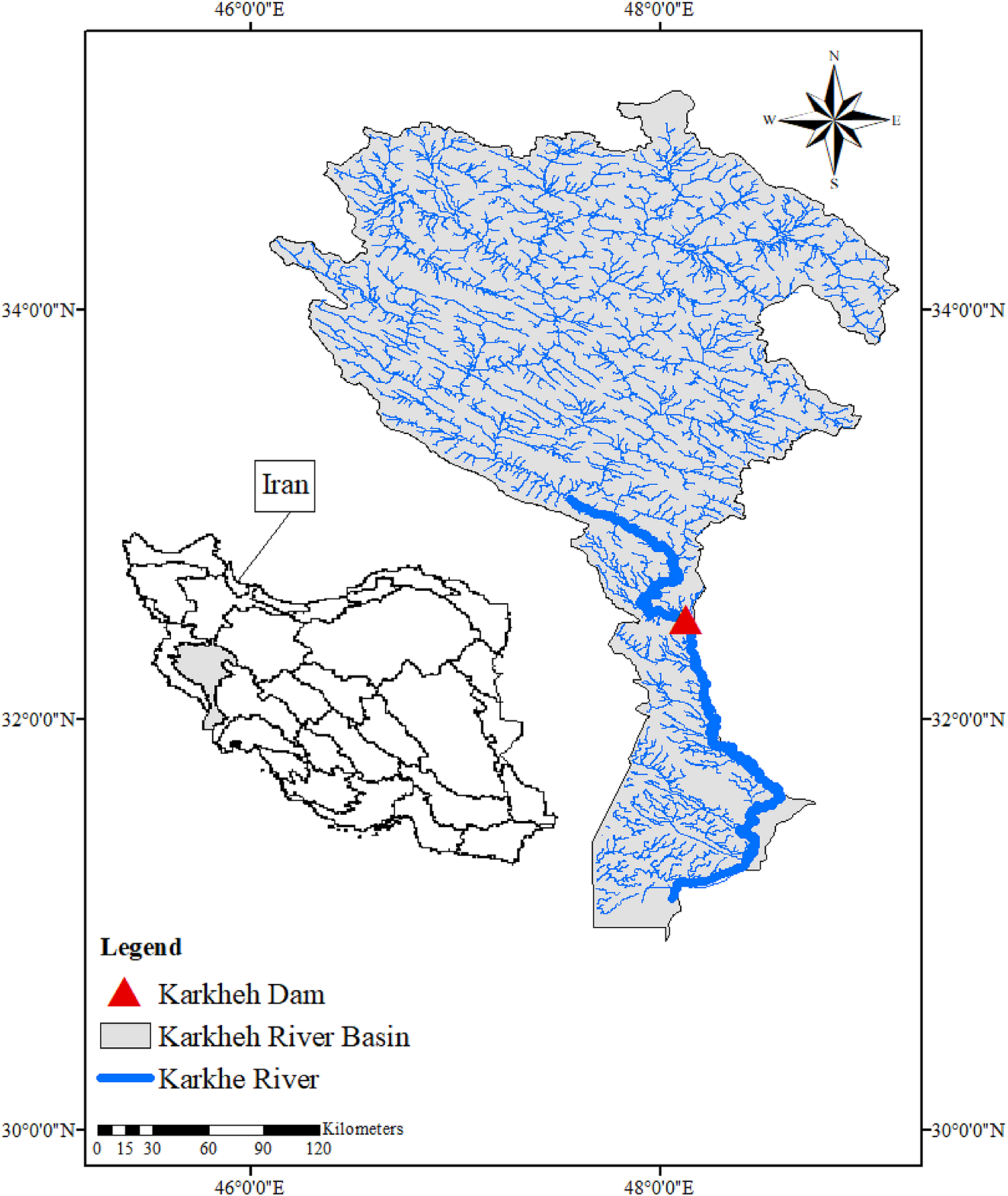
Study area and the location of Karkheh Dam. (ArcGIS 10.7.1, https://www.esri.com/en-us/arcgis/products/arcgis-pro/overview).

Due to the considerable potential of the basin in terms of renewable energy resources, many new hydropower projects have been implemented to actively expand the basin’s capacity to generate electricity^19^. The Karkheh Dam, with an installation capacity of 400 MW, is among one of the major infrastructures that has been in an operational stage since 1999 to help meet the drinking, industrial, agricultural, and energy generation demands of the region. Figure 4 illustrates the Karkheh River basin and the location of the dam. The characteristics of the Karkheh Dam are shown in Table 1.

**Table 1:** Technical characteristics of the Karkheh Dam.

Normal water level (m.a.s.l.)	220
Inactive water level (m.a.s.l.)	160
Normal storage (MCM)	5347
Inactive storage (MCM)	397
Installation capacity (MW)	400
Plant factor	0.17
Power plant efficiency	0.925
Current condition	Under operation
Target users	Domestic; industry; agriculture; hydropower

As mentioned earlier, any infrastructure with a long-life expectancy, such as hydropower systems, must take into account the potential impacts of a changing climate. In this study, three *representative concentration pathway* (RCP)-based climate change projections, including RCP 2.6, RCP 4.5, and RCP 8.5, are considered. These scenarios are identified by their approximate radiative forcing (RF) values. Emission scenario RCP 2.6 peaks at 3.0 W/m^2^ and then declines to 2.6 W/m^2^ in the year of 2100; RCP 4.5 stabilizes after 2100 at 4.2 W/m^2^, and RCP 8.5 reaches 8.3 W/m^2^ in 2100 on a rising trajectory^44^. The primary objective of these scenarios is to provide all input variables necessary to run comprehensive climate models in order to reach a target RF^45^. Furthermore, to generate a local climate change trajectory, one needs to downscale the projections of a *global* or *regional circulation model*. Indeed, the model selection and downscaling technique may have a tremendous effect on the outcome of these regional climate change trajectories^19^. To carefully address this issue, this study employs three different approaches to obtain the downscaled regional climate change projections. For the first and second trajectories, the outcomes of the second generation Canadian Earth System Model (CanESM2), a global circulation model (GCM), were respectively downscaled by using the *change factor* technique and *the statistical downscaling model* (SDSM). For the third trajectory, the results from the Coordinated Regional Downscaling Experiment (CORDEX), a regional climate model (RCM), were bias-corrected by using the *quantile mapping* (QM) method. Then, the results were averaged out for each of the selected climate change scenarios (i.e., RCP 2.6, RCP 4.5, and RCP 8.5). For more information on the process of generating these regional climate change projections, readers can refer to Zolghadr-Asli et al.^19^ and Enayati et al.^46^.

**Figure 5:**
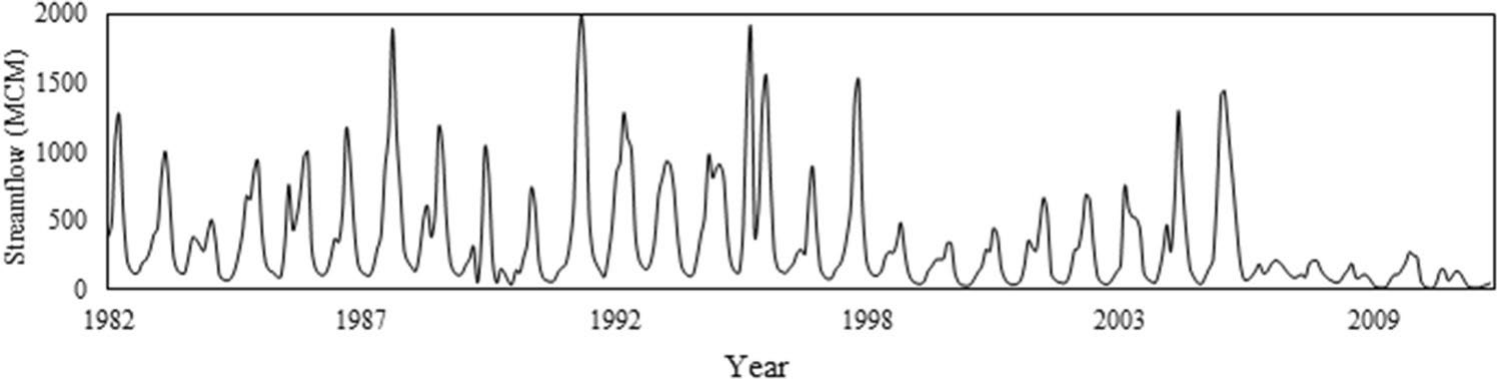
Observed streamflow time series under the *status quo* (1982–2011).

**Figure 6:**
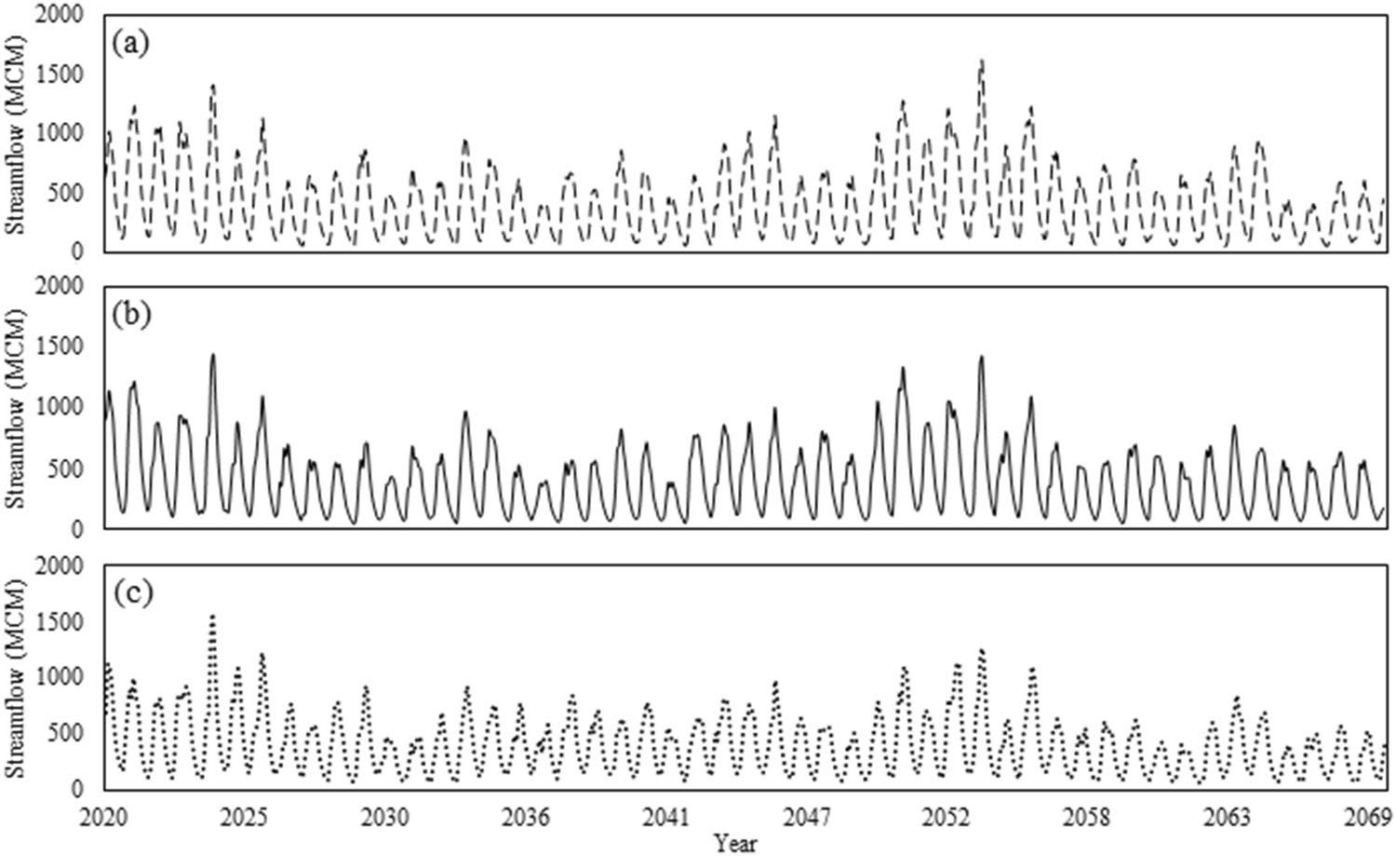
Projected streamflow time series under three climate change conditions (2020–2069): (**a**) RCP 2.6, (**b**) RCP 4.5, and (**c**) RCP 8.5.

**Figure 7:**
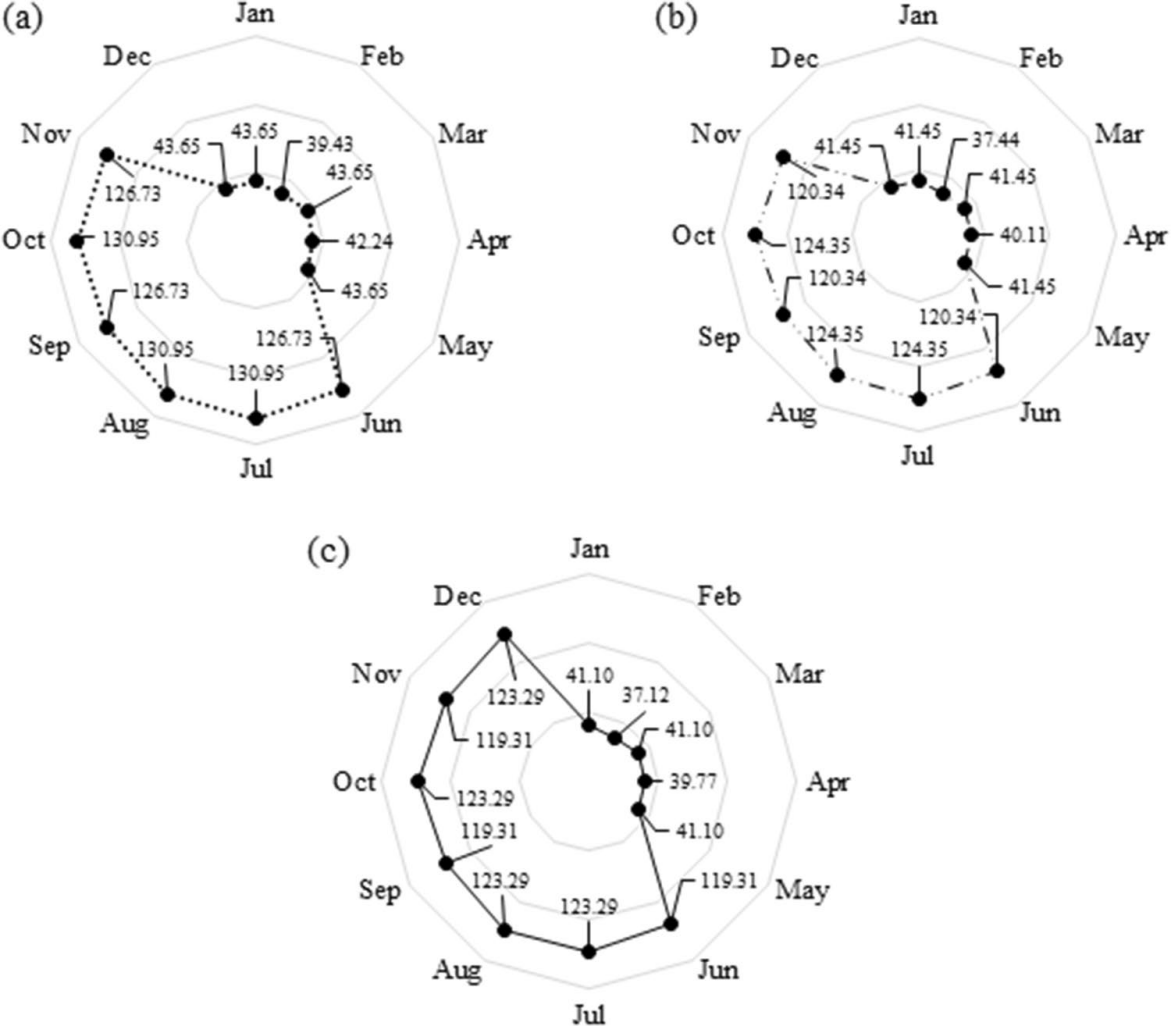
Estimated environmental demands under climate change conditions (10^6^ × m^3^): (**a**) RCP 2.6, (**b**) RCP 4.5, and (**c**) RCP 8.5.

**Figure 8:**
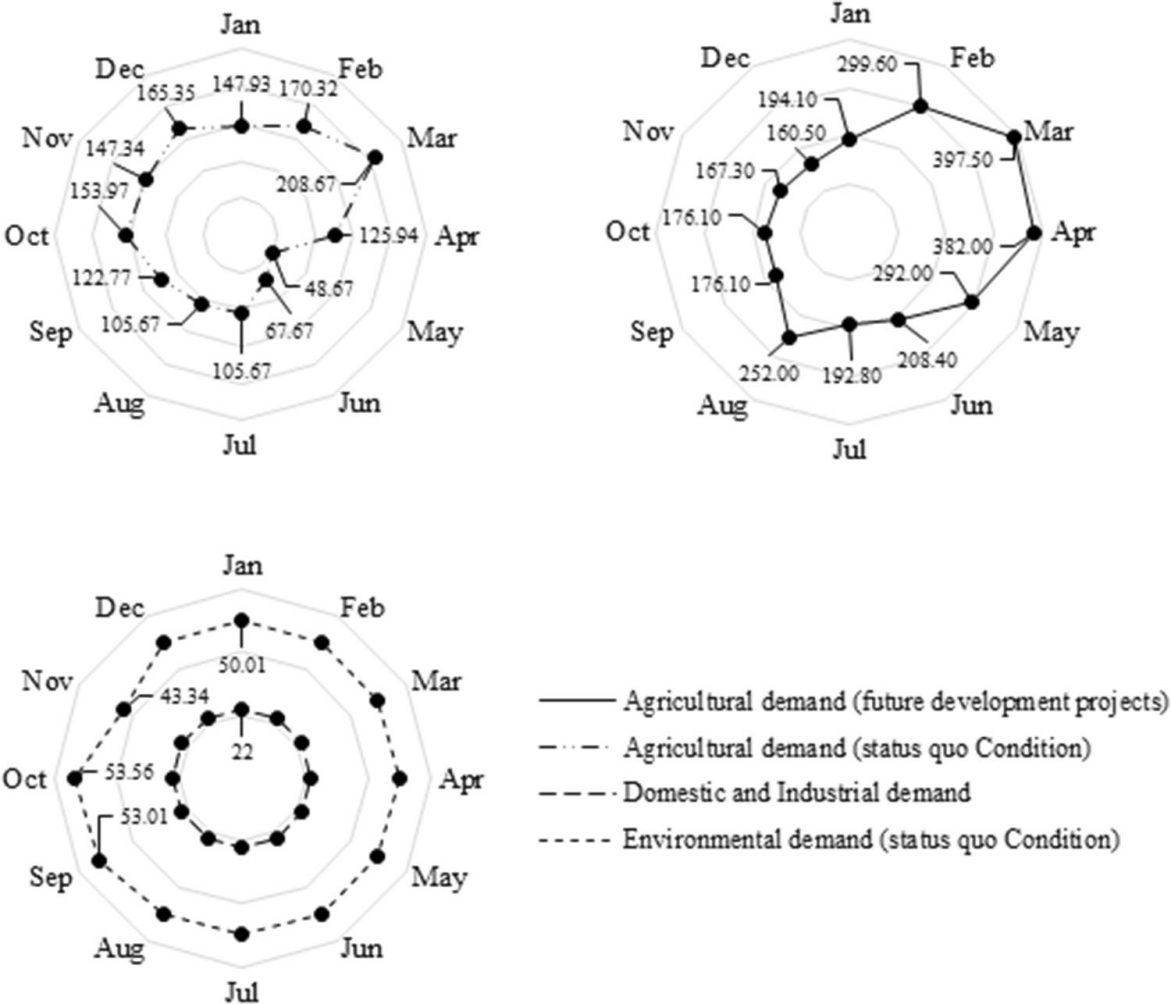
Karkheh Dam’s downstream water demands (10^6^ × m^3^).

The observed and predicted hydrographs of the Karkheh River under the *baseline condition* (1982–2011) and the three *climate change conditions* (2020–2070) are respectively shown in Figs. 5 and 6. Given the nature of this study, a fair estimation of the environmental demand is necessary to adequately address the multi-dimensionality of water resources planning and management. To that end, the *fair principle* of the *Montana method*^47^ was employed to estimate the environmental demands under the three different climate change conditions (Fig. 7). According to this principle, 10% of the mean annual flow would be set as the environmental demand in wet seasons, while in dry seasons it would increase to 30% of the mean annual flow. Accordingly, this method can provide reasonable estimations for environmental demands^48–50^. Figure 8 illustrates the remaining downstream demands including the domestic and industrial demands, and the environmental and agricultural demands under the baseline condition, and the agricultural demands based on the future development projects^18^. At this juncture, it is necessary to recap the importance of projecting the impacts of climate change on all the pillars of the WEF nexus. As demonstrated herein, in addition to the changing patterns in hydro-climatic variables that could in turn influence the status of the involved parties, climate change could also directly have an impact on these sectors. As such, a robust decision-making paradigm for water resources planning and management should be flexible enough to account for all these changes.

## Results and discussion

Inspired by the decision hierarchy depicted in Fig. 2 the main components of the current MADM problem were selected. Resultantly, the mega decision matrix of this problem was composed of 56 evaluation criteria and 27 feasible alternatives (Table 2). In this study, the environmental attributes measured how the system was able to meet the environmental water demands using a performance criterion. Similarly, the social, food, and energy attributes showed how successful the system satisfied the water demands for the municipal, agricultural, and hydropower sectors, in terms of the performance criteria. Lastly, the cost-effect measures were used to access the quantifiable economic attribute.

**Table 2:** Components of the MADM problem.

Alternatives				Attributes				
Symbol	Description	Symbol	Description	Status quo condition	Climate change conditions			Description
					RCP 2.6	RCP 4.5	RCP 8.5	
A1	PF = 0.17; PPC = 400 (MW)	A15	PF = 0.17; PPC = 540 (MW)	C1	C15	C29	C43	Reliability–social (%)
A2	PF = 0.17; PPC = 410 (MW)	A16	PF = 0.2; PPC = 400 (MW)	C2	C16	C30	C44	Reliability–environment (%)
A3	PF = 0.17; PPC = 420 (MW)	A17	PF = 0.2; PPC = 410 (MW)	C3	C17	C31	C45	Reliability–agriculture (%)
A4	PF = 0.17; PPC = 430 (MW)	A18	PF = 0.2; PPC = 420 (MW)	C4	C18	C32	C46	Reliability–hydropower (%)
A5	PF = 0.17; PPC = 440 (MW)	A19	PF = 0.2; PPC = 430 (MW)	C5	C19	C33	C47	Resiliency–social (%)
A6	PF = 0.17; PPC = 450 (MW)	A20	PF = 0.2; PPC = 440 (MW)	C6	C20	C34	C48	Resiliency–environment (%)
A7	PF = 0.17; PPC = 460 (MW)	A21	PF = 0.2; PPC = 450 (MW)	C7	C21	C35	C49	Resiliency–agriculture (%)
A8	PF = 0.17; PPC = 470 (MW)	A22	PF = 0.2; PPC = 460 (MW)	C8	C22	C36	C50	Resiliency–hydropower (%)
A9	PF = 0.17; PPC = 480 (MW)	A23	PF = 0.23; PPC = 400 (MW)	C9	C23	C37	C51	Vulnerability–social (%)
A10	PF = 0.17; PPC = 490 (MW)	A24	PF = 0.23; PPC = 410 (MW)	C10	C24	C38	C52	Vulnerability–environment (%)
A11	PF = 0.17; PPC = 500 (MW)	A25	PF = 0.23; PPC = 420 (MW)	C11	C25	C39	C53	Vulnerability–agriculture (%)
A12	PF = 0.17; PPC = 510 (MW)	A26	PF = 0.23; PPC = 430 (MW)	C12	C26	C40	C54	Vulnerability–hydropower (%)
A13	PF = 0.17; PPC = 520 (MW)	A27	PF = 0.23; PPC = 440 (MW)	C13	C27	C41	C55	Economic evaluation (%)
A14	PF = 0.17; PPC = 530 (MW)			C14	C28	C42	C56	Average annual energy produced (GW.H)

The majority of these criteria are the probability-based performance criteria (e.g., C1–C13), providing a glance at the technical aspect of these feasible renovation plans. The other notable technical criterion is the average annual generated electricity (GW.H) (e.g., C14). It should be noted that, besides the *status quo*, all these criteria are measured and considered under the three different climate change conditions (i.e., RCP 2.6, RCP 4.5, and RCP 8.5). While most of these criteria are *positive*, some criteria that are used to measure *vulnerability* (e.g., C9, C10, C11, and C12) are technically referred to as *negative* criteria. As for the alternatives, there are 27 feasible options, each of which represents a different renovation setting for the hydropower system. The first alternative (A1), for instance, depicts a situation in which the power plant’s installation capacity (PPC) would be 400 MW while the plant factor (PF) would be set to 0.17.

After a series of extensive simulations, all the evaluation criteria for the 27 feasible alternatives were measured. Next, using Shanon’s Entropy, a set of weights were determined and assigned to the evaluation criteria (Fig. 9), which were obtained by an objective approach such as Shannon’s Entropy to reflect their relative importance to the decision-maker. The higher the weights, the more crucial their roles in the selection process. The analysis of the assigned weights unveiled a consistent pattern for all of the climate change conditions (i.e., RCP 2.6, RCP 4.5, and RCP 8.5). Presumably, the weights assigned to the *social* probabilistic performance criteria (i.e., reliability, resiliency, and vulnerability) were zeros, indicating that, objectively, these criteria had the lowest priority in the decision-making process. The *status quo*, however, portrayed a different situation. With respect to the RCP 2.6 and RCP 8.5 conditions, and the *status quo*, the top-priority criterion was the *vulnerability of the energy sector*; and the computed weights for this top criterion under these three conditions were, respectively, 0.110, 0.131, and 0.027. As for RCP 4.5, the criterion with the highest priority was the *resiliency of the energy sector*, with the assigned weight of 0.070.

**﻿Figure 9:**
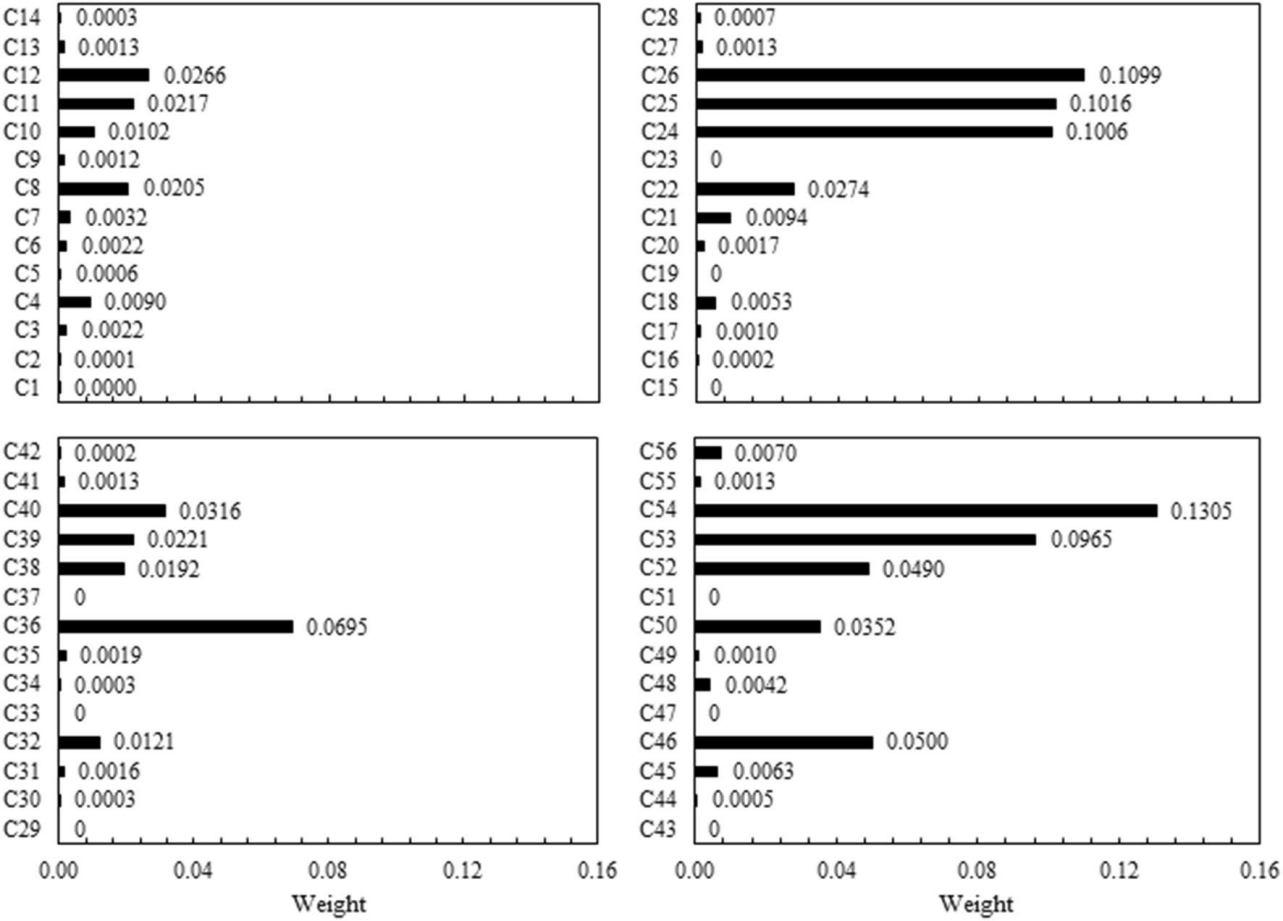
Weights assigned to the evaluation criteria.

**Figure 10:**
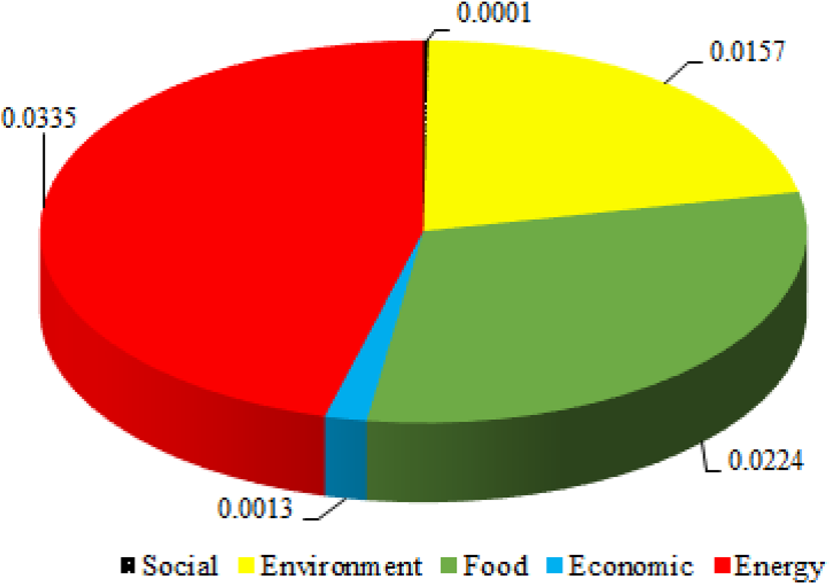
Average weights assigned to the evaluation criteria based on different aspects of the project.

Figure 10 shows the average weights assigned to the evaluation criteria based on different aspects of the project (i.e., economy, energy, environment, social, and food). Accordingly, the energy, food, and environment sectors, with the average weights of 0.034, 0.022, and 0.016, respectively, are *objectively* more involved in the decision-making process. The results revealed that the role of the social aspect of this project, on the other hand, is practically negligible.

**Figure 11:**
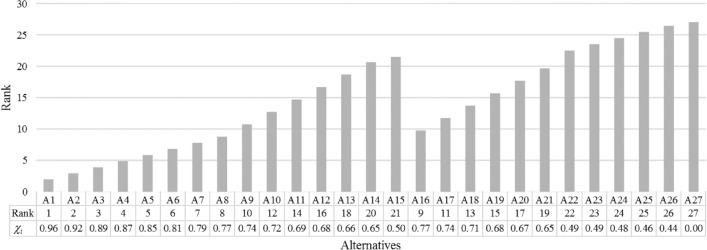
Objective ranks of the feasible alternatives.

Next, the TOPSIS method was used to rank the feasible alternatives to determine their relative desirability based on the weighted evaluation criteria. The results of this objective evaluation are displayed in Fig. 11. The first alternative (A1) emerged as the most *robust solution*, implying that if all crucial factors in the WEF security nexus are evaluated under the uncertain climate change projections, the first alternative would be selected, objectively, as the most desirable solution. From a statistical point of view, however, it should be noted that the relative closeness to the ideal solution (*χ*_*i*_), which is used to rank the alternatives, indicates that A1 (*χ*_1_ = 0.96), A2 (*χ*_2_ = 0.96), and A3 (*χ*_3_ = 0.96) may not be distinctively different, and thus these three solutions can all be labeled as robust ones. While these three alternatives can dominate other plausible solutions in terms of robustness, further in-depth assessments may be required to determine the ultimate unique order of the undominated alternatives.

It also should be noted that the desirability of the alternatives is sensitive to the variations in the plant factor (PF) variable (Fig. 11). The first, sixteenth, and twenty-third alternatives (i.e., A1, A16, and A23) are all set to have a PPC value of 400 MW, while their PF values are 0.17, 0.20, and 0.23, respectively. The overall ranks of these three alternatives are 1st, 9th, and 23rd, respectively, which suggests that the ranking is highly sensitive to the PF variable and less sensitive to the PPC variable. This notion indicates that increasing the PF of the system, while other factors remain the same, may decrease the overall performance of the system while it might seemingly be in favor of the energy sector.

## Concluding remarks

From the perspective of water resources management, a sound, sustainable, and long-term plan should reflect the notion that water is an integrated part of the WEF security nexus. The strategic role of water in a modern society imposes complex challenges to the decision-makers. According to the requirements of the WEF security nexus, most, if not all, major decisions concerning water resources planning and management need to not only account for all the pillars of the WEF nexus, but also address the environmental, socioeconomic, and technical aspects. Meanwhile, the integrity of decisions made within that platform hinges on the hydro-climatic drivers, which are, by nature, exposed to some degree of uncertainty. *Climate change* is one of the sources for this type of uncertainty, which projects different plausible futures for hydro-climatic drivers, and in turn, the associated hydrological responses. Thus, climate change can alter the perceived historical patterns of hydro-climatic variables. More importantly, this phenomenon can also have major direct or indirect impacts on energy and food—two other pillars of the WEF nexus. As such, for water-related infrastructures, especially those with long-lasting economic lifetimes such as hydropower systems, their performance can be significantly affected by these projected changes. A *robust* project, in terms of water resources planning and management, can be interpreted as a *sustainably* developed infrastructure that would not only take the multi-dimensionality of water resources into account but also can maintain a certain degree of acceptable performance under the projected fluctuations in the system inputs.

This study used this interpretation of robustness to promote an objective decision support framework (i.e., a fusion of TOPSIS and Shannon’s Entropy) to explore further the notion of a robust decision-making paradigm for water resources planning and management within the context of WEF security nexus, which also accounts for the uncertainties imposed by the climate change phenomenon. Derived from an objective point-of-view, this framework provides a pragmatic perspective to opt for a robust solution that considers the environmental, socioeconomic, and technical factors; and more importantly, with minor computational adjustment it can assess even more evaluation criteria and/or feasible alternatives. To demonstrate the applicability of this framework, the Karkheh River basin in Iran was selected as a case study. Using a set of environmental, socioeconomic, and technical attributes, the most robust renovation plan for the Karkheh Dam was identified for both *status quo* and *climate change* conditions. As the robust solution, the system has a power plant capacity of 400 MW and a plant factor of 0.17. Accordingly, the renovated system can secure the WEF nexus for the next 50 years while tolerating the potential climatic changes in the region. While increasing the power plant capacity or plant factor would be, seemingly, in favor of the energy sector, if all relevant factors are to be considered, the overall performance of the system might become sub-optimal as a result of such changed settings.

## Data Availability

The data that support the findings of this study are available from the corresponding author upon reasonable request.
